# Machine Learning-Assisted Multi-Property Prediction and Sintering Mechanism Exploration of Mullite–Corundum Ceramics

**DOI:** 10.3390/ma18061384

**Published:** 2025-03-20

**Authors:** Qingyue Chen, Weijin Zhang, Xiaocheng Liang, Hao Feng, Weibin Xu, Pengrui Wang, Jian Pan, Benjun Cheng

**Affiliations:** 1 School of Energy Science and Engineering, Central South University, Changsha 410083, China; 2 School of materials and metallurgy, University of Science and Technology Liaoning, Anshan 114051, China; 3 School of Minerals Processing and Bioengineering, Central South University, Changsha 410083, China

**Keywords:** mullite–corundum ceramics, machine learning, ceramics properties, experimental validation, sintering mechanism

## Abstract

Mullite–corundum ceramics are pivotal in heat transfer pipelines and thermal energy storage systems due to their excellent mechanical properties, thermal stability, and chemical resistance. Establishing relationships and mechanisms through traditional experiments is time-consuming and labor-intensive. In this study, gradient boosting regression (GBR), random forest (RF), and artificial neural network (ANN) models were developed to predict essential properties such as apparent porosity, bulk density, water absorption, and flexural strength of mullite–corundum ceramics. The GBR model (*R*^2^ 0.91–0.95) outperformed the RF and ANN models (*R*^2^ 0.83–0.89 and 0.88–0.91, respectively) in accuracy. Feature importance and partial dependence analyses revealed that sintering temperature and K_2_O (~0.25%) positively affected bulk density while negatively influencing apparent porosity and water absorption. Additionally, sintering temperature, additives, and Fe_2_O_3_ (optimal content ~5% and 1%, respectively) were positively related to flexural strength. This approach provided new insight into the relationships between feedstock compositions and sintering process parameters and ceramic properties, and it explored the possible mechanisms involved.

## 1. Introduction

Mullite–corundum ceramics are of particular interest due to their superior mechanical strength, excellent thermal stability, and robust resistance to chemical corrosion [1,2,3,4,5]. The performance properties of mullite–corundum ceramics make them particularly suitable for high-temperature and high-pressure applications, such as CSP (concentrated solar power) heat transfer pipelines and thermal energy storage systems [6]. High bulk density contributes to structural stability and improved thermal conductivity, which are essential for withstanding extreme conditions [7,8]. Low water absorption and porosity enhance resistance to chemical corrosion and infiltration of thermal media, ensuring durability in environments involving gaseous or liquid heat transfer mediums. Additionally, reduced apparent porosity minimizes thermal losses, increasing energy efficiency, while high flexural strength ensures reliability under significant mechanical and thermal stresses, establishing these ceramics as a highly viable material for demanding industrial applications [9,10]. Prior research has studied the properties of mullite–corundum composite materials through extensive experiments [11]. However, optimizing the properties of corundum-mullite ceramics through traditional experimental methods presents significant challenges [8,12]. The iterative nature of experimental trials is both time-consuming and expensive, often requiring extensive testing to achieve the desired material characteristics. To address the limitations of traditional experiments, there is a need for a more effective approach. Machine learning (ML) provides a promising alternative, enabling the identification of key relationships between raw materials and manufacturing parameters and material properties, thus reducing the need for extensive physical testing.

ML has recently been adopted in various material research contexts to uncover hidden relationships among diverse parameters, such as composition, properties, and process variables, by analyzing large experimental datasets [13,14,15]. By leveraging large datasets and advanced algorithms, machine learning can identify complex patterns and relationships that are not easily discernible through traditional methods [16]. ML has been widely utilized in ceramics domains. For example, various properties of conventional and advanced ceramics, such as thermal conductivity, bending strength, hardness, ballistic performance, and melting temperature, can be predicted using machine learning methods [17,18,19,20,21]. Similarly, the prediction of structural Si_3_N_4_ ceramic strength and high-entropy ceramics properties including Young’s modulus, hardness, and wear resistance values were accurately assisted by different ML models such as extreme gradient boosting (XGBoost), random forest (RF), support vector regression (SVR), artificial neural network (ANN), etc. [22,23,24].

In addition, the ML model (RF, SVR, K-nearest neighbors (KNN), and back propagation neural networks (BPNN)) was established based on few-shot datasets to predict the bulk density and cold compressive strength performance of Al_2_O_3_-SiO_2_ porous ceramics, which were also verified by experiments [25]. Particularly, the optimal RF, gradient boosting regression (GBR), and ANN models were also developed to predict the properties of ceramic matrix composites, exhibiting superior prediction metrics, remarkable accuracy, and robustness [26,27]. These three models were particularly effective at handling nonlinear interactions between variables, making them ideal for predicting complex ceramic properties [27,28,29,30].

ML models facilitated the interpretation of complex mechanisms by uncovering hidden relationships between input parameters and material behavior, with feature importance and dependence analysis further enhancing the understanding of these interactions in materials and structures [31,32]. Dimensionality reduction methods, integrated with ML models, enhanced performance by reducing data complexity and improving model interpretability, as demonstrated in multi-walled carbon nanotube deformation predictions [33]. Additionally, shapely additive explanations (SHAP) have been employed to assess the contribution of various design parameters in composite beam structures, providing deeper insights into how these parameters affect material behavior [34]. A comparison of different feature importance measures in classification models has also revealed their critical role in identifying and quantifying the most influential parameters [35]. Although ML-based predictions have been applied to accurately predict the properties of ceramic materials, there is room to enhance the interpretability of these models. Notably, despite the progress in predicting specific ceramic properties, few studies have addressed the prediction of multiple distinct fundamental properties of mullite–corundum ceramics, highlighting a gap in the current research landscape.

In this study, ML was employed to predict multiple fundamental properties by considering chemical compositions (Al_2_O_3_, SiO_2_, MgO, K_2_O, CaO, TiO_2_, Fe_2_O_3_, Na_2_O, additive, and others) and sintering process parameters (sintering temperature, sintering time, and heating rate) as inputs, with bulk density, apparent porosity, water absorption, and flexural strength as outputs. In detail, four single-target predictive models for the properties of a mullite–corundum ceramic were established. The ML-based importance and feature analyses for each prediction target were conducted to investigate the relationship between input and output targets. Three ML algorithms, GBR, RF, and ANN, were compared and optimized to achieve accurate prediction of single-target tasks. Moreover, the impacts of various features on the mullite–corundum ceramic were analyzed based on the optimal ML models. Based on the predicted results, a possible sintering mechanism was proposed to further elucidate the effect of input variables on ceramic properties. Finally, experimental validation was conducted to support the accuracy of the models in predicting mullite–corundum ceramic properties.

## 2. Methods

### 2.1. Dataset Acquisition and Pre-Processing

The dataset about properties of mullite–corundum ceramic materials was compiled from 22 published papers in the Elsevier and Web of Science databases. The inputs contained 13 features, including chemical compositions such as Al_2_O_3_ (%), SiO_2_ (%), MgO (%), Fe_2_O_3_ (%), CaO (%), TiO_2_ (%), K_2_O (%), Na_2_O (%), other undetected minor oxides (Equation (1)) and additives (%) (such as MgO, Sm_2_O_3_, Tm_2_O_3_, etc.), as well as sintering processing parameters (e.g., sintering temperature, sintering time, and heating rate). The apparent porosity (%), bulk density (g/cm^3^), water absorption (%), and flexural strength (MPa) were considered as outputs. A total of 495 data samples were screened and divided into four sub-datasets: (i) 344 data samples for apparent porosity (dataset #1), (ii) 440 data samples for bulk density (dataset #2), (iii) 362 data samples for water absorption (dataset #3), and (iv) 413 data samples for flexural strength (dataset #4).

Others = 100 − (Al_2_O_3_ + SiO_2_ + MgO + Fe_2_O_3_ + CaO + TiO_2_ + K_2_O + Na_2_O) (1)

All units of each feature were unified, and the sum of the chemical compositions from inputs was 100%. The input features were standardized using Equation (2), which minimized redundancy bias, improved data consistency, and optimized database efficiency [36].

(2) $$Z_{s}=\frac{\left(Z_{i}^{*}-\bar{Z}\right)}{\sigma }$$

where $Z_{i}^{*}$ is the *i*th experimental value; $\bar{Z}$ and σ indicate the mean value of the experimental values and standard deviation, respectively.

Pearson correlation analysis was conducted to preliminarily explore the relationships between input and output targets [37]. The Pearson correlation coefficient (PCC, *r* value) was calculated by Equation (3).

(3) $$r_{xy}=\frac{\sum_{i=1}^{n}\left(x_{i}-\bar{x}\right)\sum_{i=1}^{n}\left(y_{i}-\bar{y}\right)}{\sqrt{\sum_{i=1}^{n}{\left(x_{i}-\bar{x}\right)}^{2}}\sqrt{\sum_{i=1}^{n}{\left(y_{i}-\bar{y}\right)}^{2}}}$$

where $r_{xy}$ is the PCC value of any two variables *x* and *y*; $\bar{x}$ and $\bar{y}$ mean the average values of *x* and *y*, respectively; *n* represents the amount of data for *x* or *y*. $x_{i}$ and $y_{i}$ are the *i*th value of *x* and *y*, respectively. The positive and negative *r* values represent the strength of the positive and negative correlation between the two variables, respectively.

Prior to developing the ML models, the pre-processed dataset was randomly partitioned, with 80% of the data allocated for the training dataset to construct the predictive models, while the remaining 20% was reserved as a testing dataset to evaluate the performance of the ML models.

### 2.2. Machine Learning Algorithms and Model Optimization

In the present work, three integrated ML algorithms, the GBR, RF, and ANN models, were employed to develop predictive regression models (Figure S1). These models were implemented based on Python (version 3.11) and the scikit-learn library (version 1.4.2). RF, a machine learning algorithm that combines decision trees, effectively handles nonlinear relationships between variables [38]. GBR, an ensemble learning algorithm, was trained by adopting a boosting strategy [39]. ANN, a data-driven modeling technique, was inspired by the human nervous system [27]. Compared to other ML models, these ML models demonstrate strong generalization and interpretability performance, fast convergence, and adaptability to complex features for moderate-sized datasets. Recent research has indicated the superior predictive performance of these algorithms in predicting ceramics’ properties, demonstrating their significant potential in this domain [40].

A 5-fold cross-validation strategy was employed to optimize two key hyper-parameters of the ML models. For the GBR and RF models, the optimal hyper-parameters, including n_estimators and max_depth, were selected based on the highest average test R^2^ achieved during cross-validation. The number for n_estimators and max_depth varied from 2 to 128, with other hyper-parameters kept at default values [41]. For the ANN model, the Relu function and Adam algorithm were selected as the activation function and learning algorithm in this work [42]. The number of neurons within the two hidden layers was tested from 1 to 100 to obtain the best-trained neural network.

### 2.3. Modeling and Evaluation

A single-target model was developed to predict the properties of mullite–corundum ceramic materials, allowing for an exploration of the impact of input features on each output target. The ML model converges by minimizing the loss function, optimizing the training parameters, and reducing prediction errors. The loss function for the single-target ($L_{S}$) predictive model was calculated using the following expression:

(4) $$L_{S}=\sum_{i=1}^{n}{\left(Z_{i}-\bar{Z_{p}}\right)}^{2}$$

where n is the number of actual values in the node, *i* represents the *i*th data point used during the training, and $Z_{i}$ indicates the *i*th actual value; $\bar{Z_{p}}$ indicates the mean value of the node and the predicted value of the child node.

Three statistical indicators were used to evaluate the models: the determination coefficient (*R*^2^), the root mean squared error (*RMSE*), and the mean absolute error (*MAE*). The formulas for calculating *R*^2^, *RMSE,* and *MAE* are provided in Equations (5)–(7), respectively.

(5) $$R^{2}=1-\frac{\sum_{i=1}^{n}{\left(Z_{i}^{*}-{\hat{Z}}_{i}\right)}^{2}}{\sum_{i=1}^{n}{\left(Z_{i}^{*}-\bar{Z}\right)}^{2}}$$

(6) $$RMSE=\sqrt{\frac{1}{n}(\sum_{i=1}^{n}{\left(z_{i}^{*}-{\hat{z}}_{i}\right)}^{2})}$$

(7) $$MAE=\frac{1}{n}\sum_{i=1}^{n}\left|Z_{i}^{*}-{\hat{Z}}_{i}\right|$$

where *n* indicates the number of test samples; $Z_{i}^{*}$ and ${\hat{Z}}_{i}$ indicate the *i*th experimental value and corresponding predicted value, respectively; $\bar{Z}$ indicates the mean value of the *n* experimental values.

### 2.4. Evaluation of Input Feature Influence on Target Variables

The three ML models, GBR, RF, and ANN, were compared in terms of prediction performance, and the model demonstrating superior performance was selected for further analysis. To elucidate the relative contributions of input features to the output targets, a feature importance analysis was conducted on the basis of the developed ML models. The relationships between crucial input and output targets were analyzed to understand the effects of input features on the targets (outputs). Therefore, one-way partial dependence plots were generated to illustrate the relationships between each vital feature and the output targets, accounting for the average effect of other model predictors [43]. In addition, the SHAP method, as a game-theoretic approach, was also widely applied to feature analysis. The residual analysis utilized the Kolmogorov–Smirnov (K–S) test (with a 95% confidence interval) to calculate the significance of residues (p_K–S) on both training and testing data in the Statistical Product and Service Solutions (SPSS) statistical software (version 26.0). The residues obey normal distribution mathematically when p_K–S > 0.05 [44], indicating excellent reliability and accuracy of ML prediction models [39]. The impact of each input feature on the predicted target was explored, revealing various types of correlations, including linear, monotonic, and complex relationships.

### 2.5. Model Application and Experimental Validation

Synthesized nanocrystalline mullite powders and commercially coarse-grained Al_2_O_3_ powders (Sinosteel Luoyang Institute of Refractories Research Co., Ltd., Luoyang, China) were employed as raw materials; the experimental schemes and chemical compositions are listed in Table 1. The experimental flowchart is illustrated in Figure S2. The schemes B0 to B3 were sourced from another study [45] to ensure the generalization and accuracy of the predictive model across various conditions. The new scheme B4 was developed and tested in this study and was based on a similar methodology and materials used in the study by Liu et al. [27], ensuring a reasonable level of consistency for the experiment. The chemical compositions were obtained by X-ray fluorescence (XRF) (Bruker, Billerica, MA, USA). The powders were uniformly blended using alumina balls within a planetary ball mill, followed by uniaxial pressing at 20 MPa in a steel die to obtain bars measuring 40 mm × 40 mm × 5 mm and sintered at different temperatures (1480, 1500, 1530, 1560, and 1580 °C) for 3 h with a heating rate of 5 °C/min. To minimize experimental uncertainty, five samples were tested to obtain an average value. The microstructure of the samples was observed by a scanning electron microscope (SEM) (Nova400NanoSEM, Amsterdam, The Netherlands). The optimized single-target prediction model was applied to predict ceramic properties under the above experimental conditions. The predicted and experimental values were then compared.

## 3. Results and Discussions

### 3.1. Statistical Analysis of the Pre-Processed Dataset

**Figure 1:**
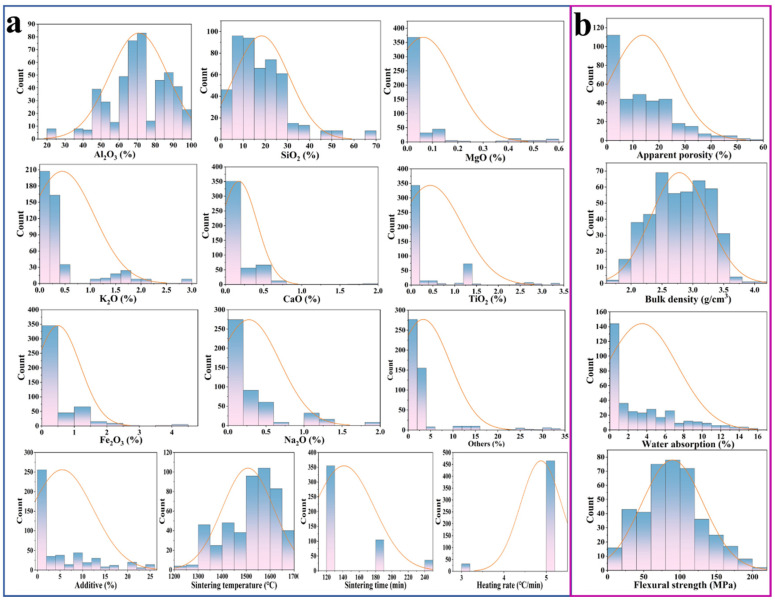
The data distribution of (**a**) chemical compositions, sintering process parameters, and (**b**) properties of mullite–corundum ceramic materials.

In Figure 1 and Table 2, the statistical distributions and variables of all input and output targets are presented, elucidating the preparation process and properties of mullite–corundum ceramics. As can be seen, Al_2_O_3_ and SiO_2_ were the main contributors to the composition, peaking around 50–70% and 10–30%, respectively. Additives and minor oxides such as MgO, K_2_O, and CaO were primarily concentrated below 1%. The sintering parameters showed a bell-shaped distribution with the temperature optimally ranging from 1500–1600 °C, while the sintering time and heating rate were predominantly maintained at 120 min and 5 °C/min, respectively. It was worth noting that the broad and diverse distributions of input variables represented in the collected dataset could improve the generalization of models in the learning process.

The statistical analysis results of output targets are shown in Figure 1. Apparent porosity and water absorption exhibited distributions peaking around 10–20% and 5–10%, respectively, while bulk density showed a normal distribution centered around 2.5 g/cm^3^, and that of flexural strength was chiefly located in 100–150 MPa. In summary, the numerical distribution ranges of apparent porosity, bulk density, water absorption, and flexural strength were 0.05–57.70%, 1.70–23.62 g/cm^3^, 0.01–15.92%, and 2.76–210.67 MPa, respectively.

To evaluate whether the considered input variables can provide valuable information for ML model development to predict mullite–corundum ceramic properties, we conducted the Pearson correlation analysis to roughly understand the correlations between inputs and outputs. The higher Pearson correlation coefficient meant a higher correlation with output, and a negative value represented a negative relationship.

**Figure 2:**
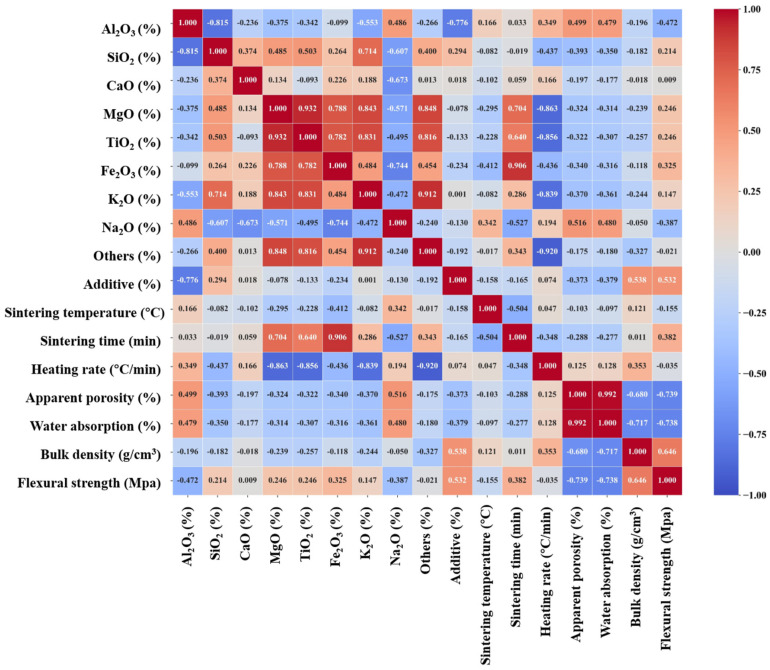
Pearson correlation coefficients (r) matrix between inputs and outputs.

According to the Pearson correlation coefficients matrix and significance level of correlation coefficients (Figure 2), the apparent porosity and water absorption had a relatively strong positive correlation with the content of Al_2_O_3_ (r = 0.50 and 0.48) and Na_2_O (r = 0.52 and 0.48). In addition, flexural strength was negatively correlated with Al_2_O_3_ (r = −0.47) and Na_2_O (r = −0.38). Al_2_O_3_ and Na_2_O might influence the formation of pores during the sintering densification process [46,47], while Na_2_O, acting as a flux, might increase porosity and water absorption if not properly controlled [48]. Both bulk density and flexural strength were also correlated with additives (r = 0.53), which may facilitate grain refinement and densification [49]. For the sake of data integrity and to avoid the potential loss of important features, this study employed datasets without dimensionality reduction for model development as a way to fully assess the impact of the collected input features on the output.

Additionally, for output targets, apparent porosity and water absorption exhibited an almost perfect positive correlation (r = 0.99), which might be due to higher porosity creating more voids, allowing for greater water absorption [50]. Conversely, both showed negative correlations with bulk density (r = −0.92 and −0.72, respectively), which can be attributed to the densification process during sintering. Initially, particle rearrangement reduced porosity; as the liquid phase filled the pores, the material underwent further densification through the bonding of particles. This led to a reduction in porosity, with the volume occupied by voids being reduced, which consequently increased bulk density [51,52]. Flexural strength appeared to be positively correlated with bulk density (r = 0.65) and negatively correlated with apparent porosity and water absorption (r = −0.74), implying that denser ceramics with lower porosity may have higher mechanical strength. However, no significant correlations were observed between the sintering parameters and the output targets, indicating the potential need for machine learning models to better identify and optimize factors affecting ceramic properties.

### 3.2. Hyper-Parameter Adjusting and Optimization

Figure S3 illustrates the predictive performance outcomes, specifically the testing *RMSE*, achieved by training models that employ a combination of five-fold cross-validation for hyper-parameter tuning. For GBR and RF models, two hyper-parameters were optimized (Table 3). The values of the *RMSE* of the GBR model showed satisfactory results when n_estimators were higher than 20, and max_depth was greater than 3 and lower than 8 (Figure S3a–d). The optimal values were a max_depth of 5 and n_estimators of 81, 75, 101, and 71 for Figure S3a, S3b, S3c and S3d, respectively. For RF (Figure S3e–h), the values of the *RMSE* were improved with the increase of max_depth and n_estimators until they reached ~9 and ~15. Therefore, moderate n_estimators (52) and relatively small max_depth (9 or 18) were chosen to avoid the overfitting of predictive models (Figure S3i–l). For the ANN model, the optimal first and second hidden layers with 60, 10, 64, and 88 and 50, 60, 64, and 64 neurons, respectively, are represented in Table S1.

Based on the hyper-parameter optimization processes described, the hyper-parameters for the ML models applied to other datasets (#1–4) with varying input variables were similarly optimized. The results of the model prediction performance are depicted in Table 4.

### 3.3. Multi-Property Prediction of Mullite–Corundum Ceramics

#### 3.3.1. Model Evaluation and Performance Analysis

**Figure 3:**
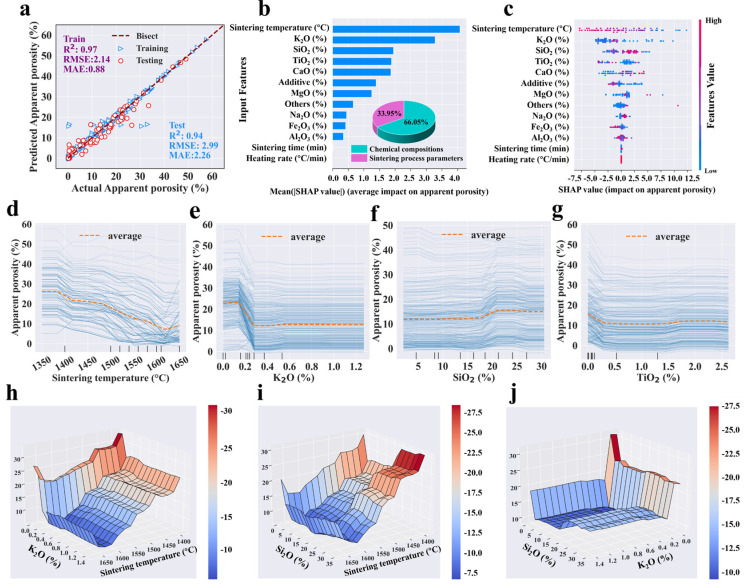
The results of the (**a**) predictive performance, (**b**) feature importance, (**c**) impact, (**d**–**g**) one-way partial dependence plots of the top four inputs, and (**h**–**j**) two-way partial dependence plots of the top three inputs to the apparent porosity prediction by the GBR model (dataset #1).

**Figure 4:**
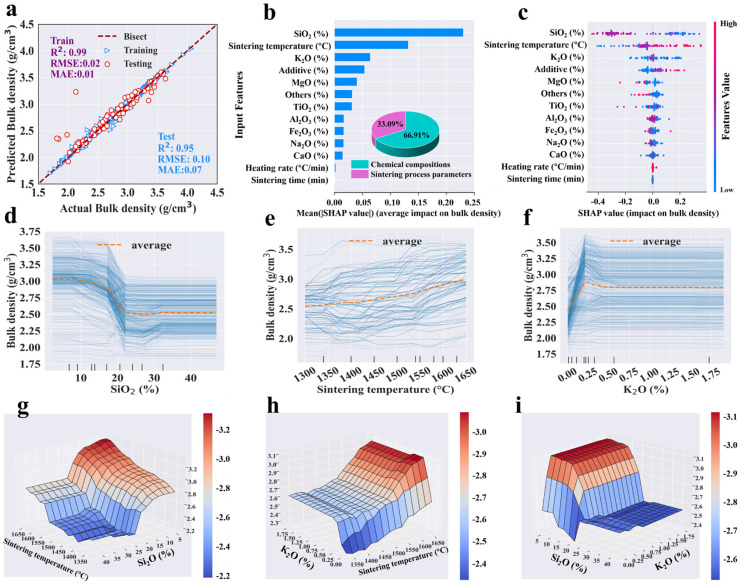
The results of the (**a**) predictive performance, (**b**) feature importance, (**c**) impact, (**d**–**f**) one-way, and (**g**–**i**) two-way partial dependence plots of top three inputs to the bulk density prediction by the GBR model (dataset #2).

**Figure 5:**
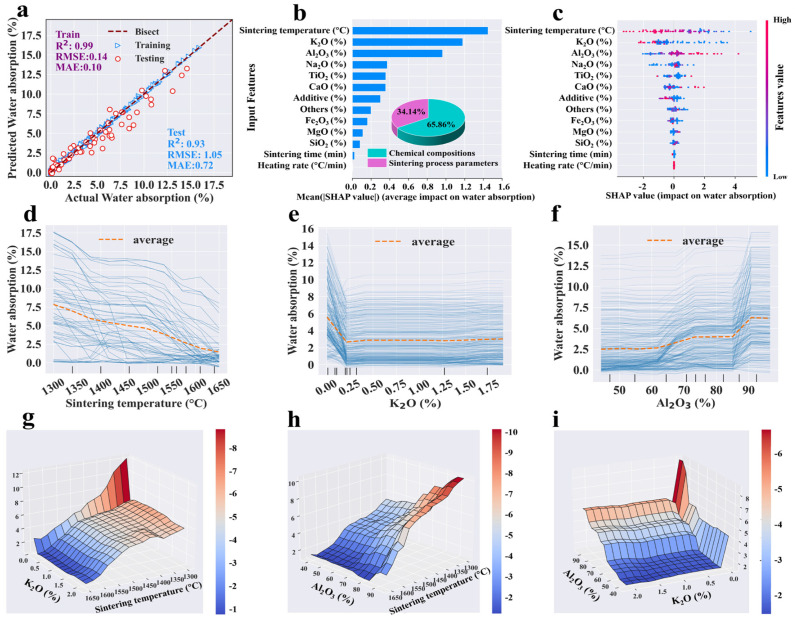
The results of the (**a**) predictive performance, (**b**) feature importance, (**c**) impact, (**d**–**f**) one-way, and (**g**–**i**) two-way partial dependence plots of top three inputs to the water absorption prediction by the GBR model (dataset #3).

**Figure 6:**
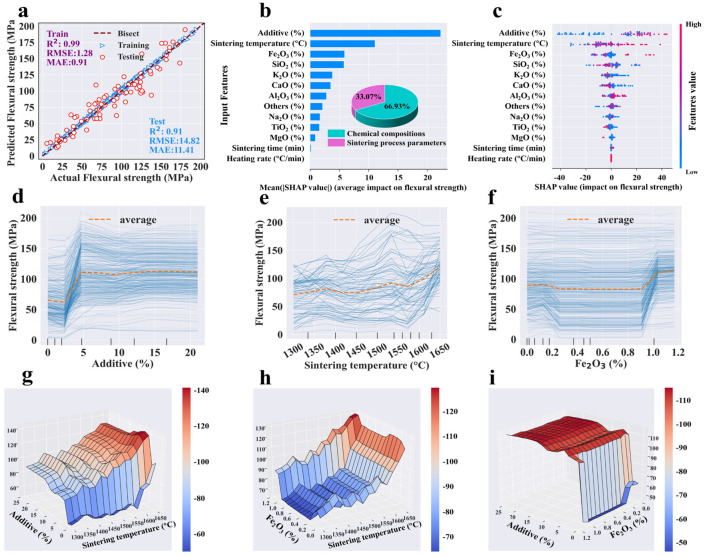
The results of the (**a**) predictive performance, (**b**) feature importance, (**c**) impact, (**d**–**f**) one-way, and (**g**–**i**) two-way partial dependence plots of top three inputs to the flexural strength prediction by the GBR model (dataset #4).

Following the hyper-parameter tuning, the entire dataset was reprocessed using the optimized hyper-parameters, and Table 3 details the performance of each single-target model, including *R*^2^, *RMSE*, and *MAE*. Given that limited data points were allocated for cross-validation during the hyper-parameter tuning process, the mean *R*^2^ of tuning cases was typically lower than in the final models. The tuning *R*^2^ was employed solely as a criterion for selecting the optimal hyper-parameters, and it behaved differently from the final models. It was evident that the GBR, RF and ANN models achieved satisfactory fitting effects, with training *R*^2^ values exceeding 0.92 and testing *R*^2^ values above 0.83. In comparing the results of the three algorithms’ modeling, GBR expressed better performance (with higher *R*^2^ and smaller *RMSE* and *MAE*) than RF and ANN for predicting each target (Table 3 and Figure S4). It was observed that nearly all actual and predicted points were clustered near the diagonal line (y = x) (Figure 3a, Figure 4a, Figure 5a and Figure 6a). The proximity of these points to the diagonal indicated higher prediction accuracy, demonstrating a strong alignment between the predicted and actual values. The confidence coefficient for both training and testing data was set at 95%, indicating a high probability that the values fall within the red or blue zones. Smaller areas of the confidence interval denoted less uncertainty in the data values. Furthermore, the training set exhibited higher distribution accuracy and density near the bisector line compared to the testing set, indicating superior predictive performance for the training dataset, which was attributable to the inherent nature of ML algorithms.

In detail, the points of bulk density were concentrated tightly around the bisection line, with the testing *R*^2^ value reaching a high of 0.95, indicating strong prediction performance (Figure 4a). Although a few data points for apparent porosity, water absorption, and flexural strength were slightly dispersed from the bisector line, their testing R^2^ values remain above 0.91, demonstrating high accuracy (Figure 3a, Figure 5a and Figure 6a). The predicted flexural strength and apparent porosity exhibited a wider distribution span compared to water absorption and bulk density, which aligns with the previous statistical analysis results (Figure 1b). Four properties represented a strong fit and generally good performance. The residual analysis indicated that both the training and testing data residuals satisfied a normal distribution, with p_K-S higher than 0.05 (Figure S5). Additionally, most errors of the four prediction targets were close to zero (Figure S5), proving the excellent performance and reliability of the models. In summary, the optimal GBR models could accurately predict four targets with high certainty.

#### 3.3.2. Feature Analysis for Each Target (Sensitivity Analysis)

According to the optimized GBR models, feature analysis determined the relative importance of input features for apparent porosity, bulk density, water absorption, and flexural strength. The importance ranking of the input features is shown in Figure 3b, Figure 4b, Figure 5b and Figure 6b, which could evaluate the contribution of input variables to the prediction target. The feature importance ranking plot and the SHAP dependence plot combinations for each output target are displayed in Figure 3c, Figure 4c, Figure 5c and Figure 6c. The relative importance of features in ML models has traditionally been assessed using the entire dataset and has often lacked transparency in explaining the workings of black-box models. The input features were placed in order of importance level from top to bottom on the vertical axis, and the horizontal axis depicted SHAP values calculated by normalized outputs. The feature values were drawn in a gradation from blue to pink, indicating low to high significance. Moreover, to figure out the influence of each specific feature category, input features were divided into two types, namely, chemical compositions and sintering process parameters of mullite–corundum ceramics.

In detail, for apparent porosity, the sintering temperature, K_2_O, SiO_2,_ and TiO_2_ were the top four most important features among input variables based on SHAP analysis (Figure 3b). Similarly, for bulk density, those features were SiO_2_, sintering temperature, and K_2_O in order of importance level (Figure 4b). Most data points of high apparent porosity values yielded positive SHAP values, which indicated that higher sintering temperature led to higher apparent porosity, as did K_2_O (Figure 3b). In terms of bulk density, SiO_2_ and sintering temperature were most important (Figure 4b).

The impacts of critical features on the target were analyzed through one-way and two-way partial dependence plots (Figure 3d–j and Figure 4d–i), which could reveal the average influence of each feature on the output target, depicted as specific linear functions. The apparent porosity indicated a persistent declining trend, dropping from roughly 26% to 7% (Figure 3d). On the other hand, bulk density rose slowly as the sintering temperature increased (Figure 4e), with the liquid phase progressively formed and the influence of capillary forces [53]. Specifically, the slight increase in apparent porosity at high sintering temperatures may have stemmed from grain growth and the formation of new pores [54], which, combined with excessive liquid phase, induced the coalescence of smaller pores into larger ones despite progressive densification [55].

**Figure 7:**
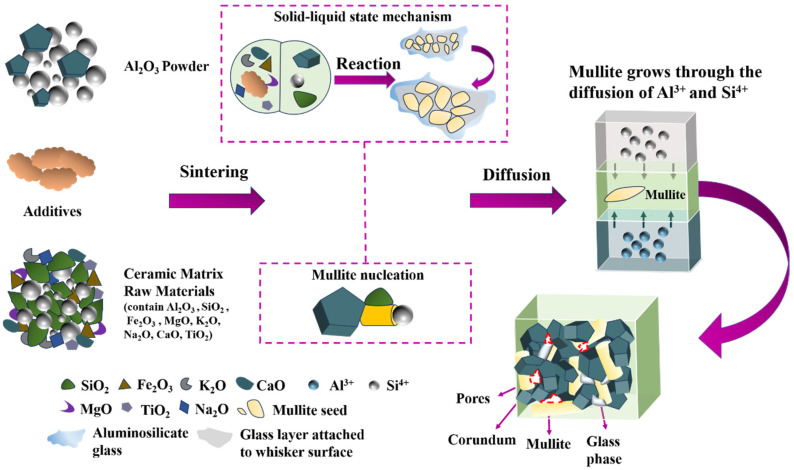
A possible schematic diagram of the sintering mechanism of mullite–corundum ceramics.

At low K_2_O levels, variability in apparent porosity and water absorption can likely be attributed to the inherent properties of the material (Figure 3e, Figure 5e and Figure S6). The preliminary increase in apparent porosity might have resulted from the enrichment of the liquid phase with K^+^ from the melting of additive, which dissolved quartz grains, increasing silica content and viscosity, thereby hindering rapid pore elimination (Figure 7) [56]. Beyond an optimal K_2_O concentration, the liquid phase reached saturation, reducing porosity and increasing bulk density [57]. Conversely, relatively high SiO_2_ content (about 20%) expanded the glass phase, increasing apparent porosity and reducing bulk density (Figure 3f and Figure 4d) [58]. The increase in TiO_2_ led to a decline in apparent porosity, likely attributable to its role in enhancing densification.

The interaction impacts of sintering temperature, K_2_O, and SiO_2_ are shown in Figure 3 and Figure 4. High sintering temperature improved apparent porosity and bulk density at high K_2_O (~0.25%) and SiO_2_ (~20%) contents (Figure 3h–j and Figure 4g–i). This was mainly owing to the grain growth, improvement of atomic diffusion, and the fluxing effects of K_2_O and SiO_2_ [59].

The top features for water absorption were sintering temperature, K_2_O, and Al_2_O_3_ in order of importance level (Figure 5b). As the sintering temperature elevated, the water absorption of the ceramic consistently diminished from about 7.8% to 1.5% (Figure 5d). Similarly, water absorption sharply decreased as K_2_O increased from 0% to 0.22%, stabilizing at 2.7% (Figure 5e). These results might be due to improved densification and reduced liquid-phase viscosity [60,61]. Therefore, the sintering temperature and K_2_O had a synergistic effect on the water absorption (Figure 5g). The water absorption value exhibited a fluctuating increase with the rise in Al_2_O_3_, as excessive Al_2_O_3_ content acted as a network modifier, increasing viscosity and hindering vitrification [62].

Similar to the previous three properties, the chemical compositions’ impact on flexural strength were twice as significant as that of the sintering process parameters. The top three features for flexural strength were additive, sintering temperature, and Fe_2_O_3_. When the additive exceeded 2.5%, the flexural strength quickly elevated by 38 MPa and then remained stable (Figure 6d). A marked increase in flexural strength was observed as the sintering temperature and Fe_2_O_3_ content rose beyond 1575 °C and 0.9%, respectively (Figure 6e). These rises might be due to the improvement of viscosity and densification, as well as stronger grain boundary bonding [11,63,64] (Figure 7). In general, additive, sintering temperature, and Fe_2_O_3_ content have a synergistic effect on flexural strength [65], as shown in Figure 6g–i. Higher temperatures improved strength through rising additive and Fe_2_O_3_ content. Therefore, the sintering temperature could be reduced by introducing additives such as conventional flux materials and rare-earth sintering additives [66,67].

A possible schematic diagram of the sintering mechanism of mullite–corundum ceramics is shown in Figure 7. The sintering mechanism may include two processes: mullite nucleation and growth. The introduction of additives and certain alkali oxides, such as K_2_O, Fe_2_O_3_, etc., may reduce the liquid-phase viscosity at high temperatures. This could potentially accelerate the diffusion of Si^4^⁺, promote the growth and grain size of mullite, and enhance the densification and physical properties of the sample [68,69,70].

### 3.4. Experimental Validation for Predictive Models

**Figure 8:**
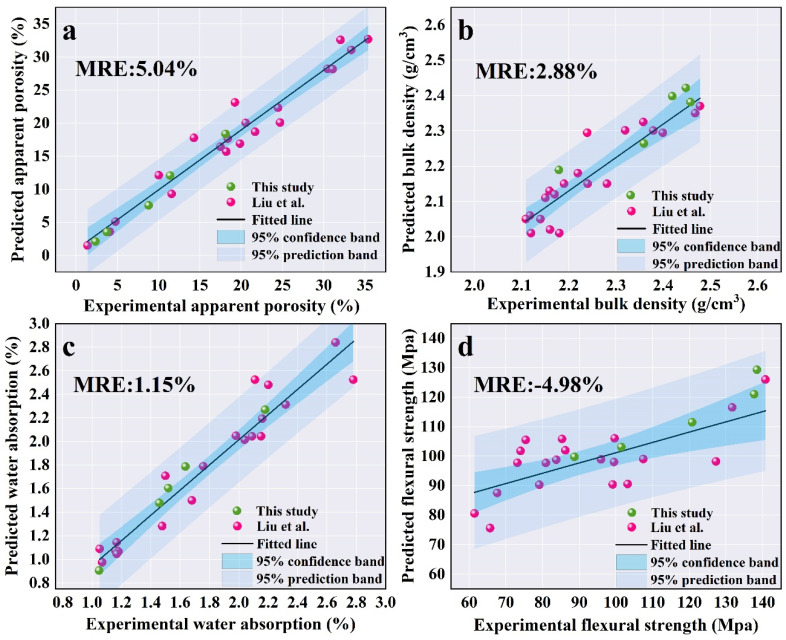
Experimental validation results of the (**a**) apparent porosity, (**b**) bulk density, (**c**) water absorption, and (**d**) flexural strength [45].

Five groups of experiments (Table S2) were utilized to validate the accuracy of the ML model in predicting the critical properties of mullite–corundum ceramics. The specifics of the experimental validation results are presented in Table S2 and Figure S6 and Figure 8, demonstrating the accuracy of the predictive models for key properties of mullite–corundum ceramics. The relative errors (Re) listed in Table S2 indicate that the predicted values closely match the experimental values, with most Re values falling within 30%, which is acceptable. In addition, most data points align well with the fitted line and fall within the 95% confidence and prediction bands, with mean relative errors (MRE) < 5.10% (Figure 8). Comparing the experimental results from this study (green points) with those from Liu et al. (red points) [45], lower errors are observed, particularly in predicting bulk density and apparent porosity, with most data points falling within the 95% confidence and prediction bands. However, larger discrepancies were observed for properties such as flexural strength, especially at higher sintering temperatures, where relative errors exceed 20% in some cases. This may be attributable to the limited data available for training and potential limitations in the model’s complexity. The combination of the table and figure demonstrates the model’s reliability while highlighting specific areas where the predictive accuracy could be enhanced.

**Figure 9:**
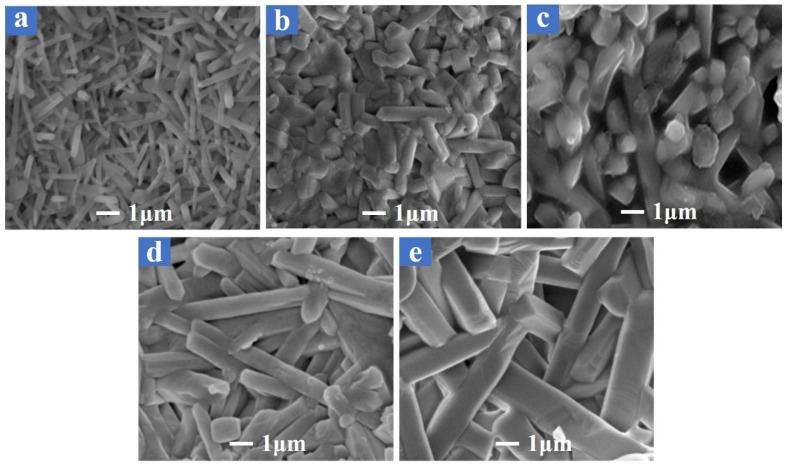
The SEM images of sample B4 sintered at different temperatures: (**a**) 1480 °C, (**b**) 1500 °C, (**c**) 1530 °C, (**d**) 1560 °C, and (**e**) 1580 °C.

Additionally, images of sample B4 sintered at different temperatures are presented in Figure 9. At 1480 °C (Figure 9a), the microstructure consisted of fine, elongated mullite grains with noticeable porosity. As the sintering temperature increased to 1500 °C (Figure 9b) and 1530 °C (Figure 9c), the grains grew larger, and the porosity reduced, consistent with the predicted decrease in apparent porosity and water absorption and the predicted increase in bulk density. At 1560 °C (Figure 9d) and 1580 °C (Figure 9e), the densification became even more pronounced, with larger, well-packed grains and minimal porosity which aligned with the model’s prediction of improved mechanical strength. The observed reduction in porosity and more tightly packed grains contributed to the increased flexural strength and reduced water absorption at these higher sintering temperatures, thereby supporting the accuracy of the model’s predictions.

## 4. Conclusions

Chemical compositions and sintering process parameters were collected to develop ML models for the prediction of multiple fundamental mullite–corundum ceramic properties, such as bulk density, apparent porosity, water absorption, and flexural strength. Three ML models with optimal hyper-parameters were successfully established, with the GBR model outperforming RF and ANN, achieving training *R*^2^ > 0.96 and testing *R*^2^ > 0.90. Feature importance and partial dependence analysis revealed that sintering temperature, K_2_O (optimal content ~0.25%), additives, and Fe_2_O_3_ (optimal content ~5% and 1%, respectively) were positively correlated with the ceramic properties, which suggested a plausible mechanism that these factors could enhance liquid-phase flow and Si^4^⁺ diffusion, facilitating mullite nucleation and growth. Experimental validation, supported by microstructural analysis, confirmed the models’ accuracy, with most predictions showing relative errors within 30%. Applying ML to mullite–corundum ceramic provided new insights into the relationships between feedstock compositions and sintering process parameters and ceramic properties.

However, this study has some limitations. The input features, including dimensions, mineralogical composition, and classification of additives, as well as additional output parameters (i.e., thermal conductivity, thermal expansion ratio, and elasticity modulus) were not considered. Incorporating these factors could provide a more comprehensive understanding of the material’s performance. Furthermore, the dataset used for ML modeling was limited. Future work should focus on expanding the dataset and incorporating additional relevant features to mitigate the adverse impact of data distribution on the ML model prediction performance and improve predictive performance.

## Figures and Tables

**Table 1:** Experimental schemes and chemical compositions.

Items	Schemes (wt %)					Chemical Compositions (%)							Ref.
	Mullite	Al_2_O_3_ Powder	Corundum	SiO_2_	Al_2_O_3_	CaO	MgO	Fe_2_O_3_	TiO_2_	K_2_O	Na_2_O	Others	
B0	100	0		38.30	53.94	–	0.04	1.51	0.65	0.54	–	5.02	[45]
B1	95	5		36.39	56.19	–	0.04	1.43	0.62	0.51	–	4.82	
B2	90	10		34.47	58.45	–	0.04	1.36	0.59	0.49	–	4.60	
B3	85	15		32.56	60.70	–	0.03	1.28	0.55	0.46	–	4.42	
B4	80		20	25.09	71.43	0.64	0.08	0.63	0.22	0.74	0.05	1.12	This study

– Data not available.

**Table 2:** Variabilities of the inputs and outputs of mullite–corundum ceramics production.

	Minimum	Maximum	Average	Standard Deviation
Al_2_O_3_ (%)	20.13	99.20	71.11	16.15
SiO_2_ (%)	0	69.73	18.17	12.48
MgO (%)	0	0.59	0.06	0.13
K_2_O (%)	0	2.81	0.44	0.63
CaO (%)	0	1.88	0.17	0.24
TiO_2_ (%)	0	3.33	0.44	0.73
Fe_2_O_3_ (%)	0	4.49	0.50	0.68
Na_2_O (%)	0	1.80	0.28	0.40
Others (%)	0	33.72	3.35	5.96
Additive (%)	0	25.74	5.49	6.89
Sintering temperature (°C)	25	1670	1493.1	166.10
Sintering time (min)	120	240	141.41	36.73
Heating rate (°C/min)	3	5	4.88	0.48
Apparent porosity (%)	0.05	57.70	13.78	12.12
Water absorption (%)	0.01	15.92	3.49	3.83
Bulk density (g/cm^3^)	1.70	4.06	2.78	0.46
Flexural strength (MPa)	2.76	210.67	89.70	42.38

**Table 3:** Results of hyper-parameter tuning for different ML models in terms of various ceramic material properties.

Dataset ^a^	Data Points	Target (Output)	ML Algorithms	Optimized Hyper-Parameters and 5-Fold Cross-Validation	
				n_Estimators	Max_Depth
#1	344	apparent porosity	GBR	81	5
			RF	52	9
#2	362	water absorption	GBR	75	5
			RF	52	9
#3	440	bulk density	GBR	101	5
			RF	52	18
#4	413	flexural strength	GBR	71	5
			RF	52	9

^a^ The input variables included chemical compositions and sintering process parameters.

**Table 4:** The summary results of the model performance of single-target ML prediction.

Dataset ^a^	Target (Output)	Optimized Model	Train *R*^2^	Train *RMSE*	Train *MAE*	Test *R*^2^	Test *RMSE*	Test *MAE*
#1	apparent porosity	GBR	0.97	2.14	0.88	0.94	2.99	2.26
	(%)	RF	0.96	2.46	1.29	0.89	3.87	2.81
		ANN	0.94	3.04	1.83	0.91	3.60	2.41
#2	water absorption	GBR	0.99	0.14	0.10	0.93	1.05	0.72
	(%)	RF	0.98	0.56	0.41	0.89	1.32	0.95
		ANN	0.93	1.01	0.64	0.88	1.39	1.00
#3	bulk density	GBR	0.99	0.02	0.01	0.85	0.18	0.09
	(g/cm^3^)	RF	0.99	0.55	0.03	0.83	0.19	0.10
		ANN	0.94	0.11	0.07	0.91	0.14	0.10
#4	flexural strength	GBR	0.99	1.28	0.91	0.91	14.82	11.41
	(MPa)	RF	0.95	9.13	6.68	0.86	18.33	13.10
		ANN	0.92	11.83	8.11	0.89	16.00	11.53

^a^ The input variables included chemical compositions and sintering process parameters.

## Data Availability

The original contributions presented in this study are included in the article/Supplementary Material. Further inquiries can be directed to the corresponding author.

## Supplementary Materials

The following supporting information can be downloaded at: https://www.mdpi.com/article/10.3390/ma18061384/s1.
