# Supplementary material for: Machine Learning-Assisted Multi-Property Prediction and Sintering Mechanism Exploration of Mullite–Corundum Ceramics
Source: Materials (Basel). 2025 Mar 20;18(6):1384. doi: 10.3390/ma18061384 (PMC11943972; doi:10.3390/ma18061384)
Supplement: Supplementary file 1 [file materials-18-01384-s001.zip › materials-3521581-supplementary.pdf]

Supplementary materials

Machine Learning-Assisted Multi-Property Prediction and Sintering Mechanism Exploration of Mullite–Corundum Ceramics

Qingyue Chen [1], Weijin Zhang [1], Xiaocheng Liang [2], Hao Feng [1], Weibin Xu [1], Pengrui Wang [2], Jian Pan [3] and Benjun Cheng [1,*]

[1] School of Energy Science and Engineering, Central South University, Changsha 410083, China
[2] School of materials and metallurgy, University of Science and Technology Liaoning, Anshan 114051, China
[3] School of Minerals Processing and Bioengineering, Central South University, Changsha 410083, China
* Correspondence: chbj666@csu.edu.cn

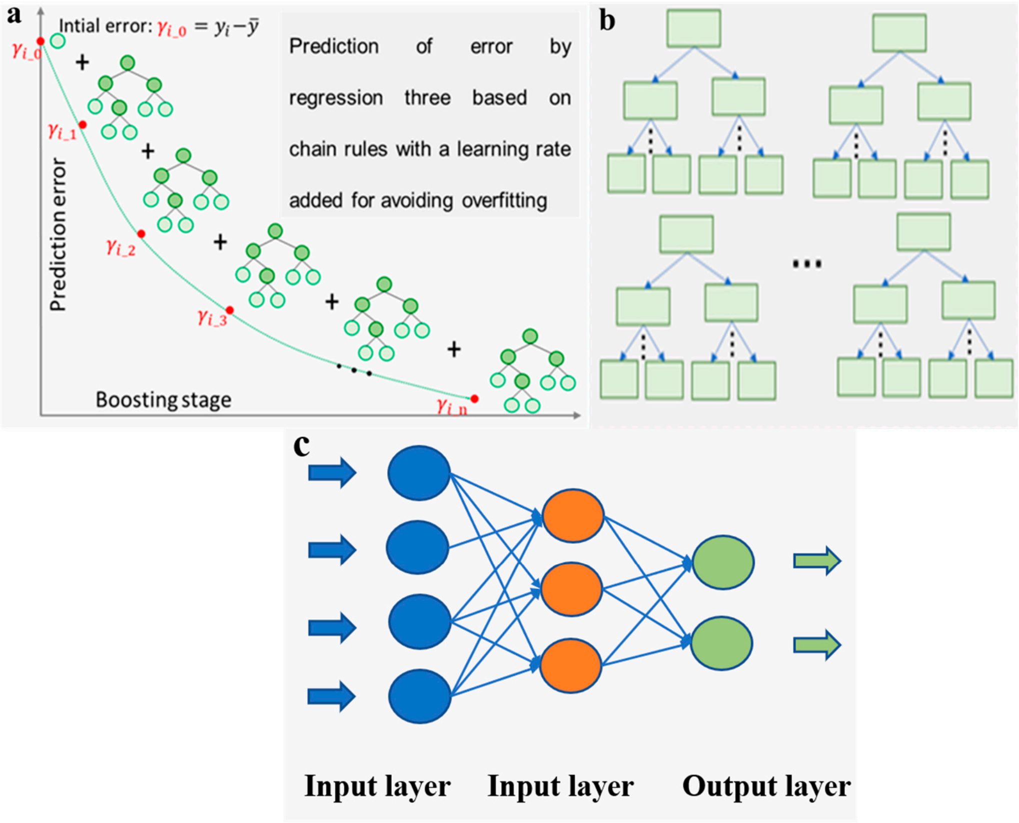

Figure S1. The algorithms of gradient boosting decision tree (a), random forest (b), and artificial neural network (ANN) model (c).

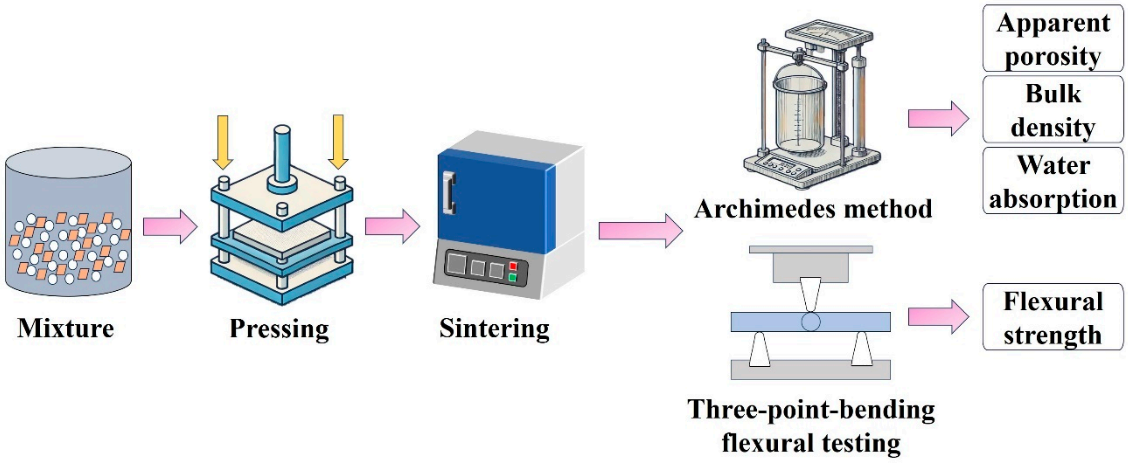

Figure S2. Experimental flowchart.

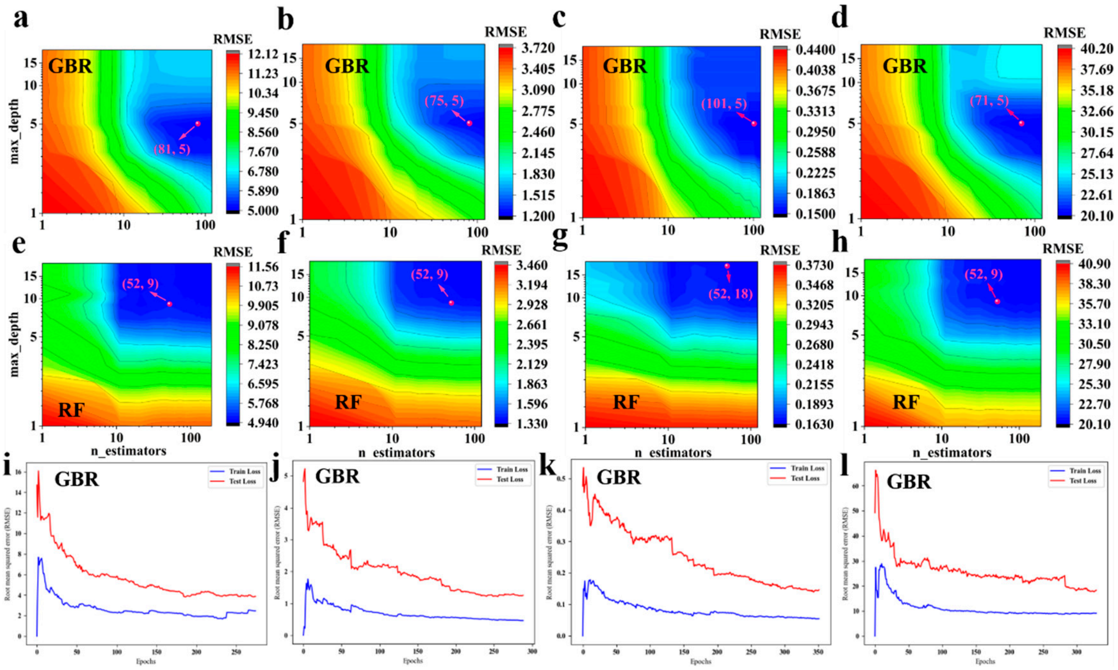

Figure S3. Hyper-parameter tuning and learning curve of ML models for predicting apparent porosity (a, e, and i), water absorption (b, f, and j), bulk density (c, g, and k), and flexural strength (d, h, and l) based on dataset #1~#4 (GBR: Gradient boosting regression, RF: Random Forest).

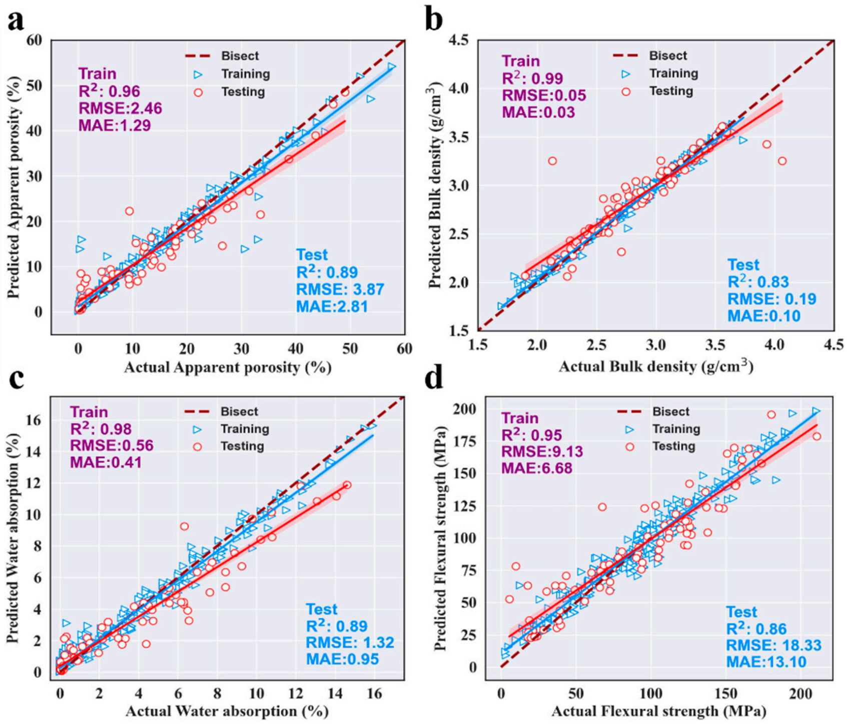

Figure S4. The performance (a-d) of optimal models for predicting apparent porosity (a) (dataset #1), water absorption (b) (dataset #2), bulk density (c) (dataset #3), and flexural strength (d) (dataset #4) of the mullite-corundum ceramic based on RF models.

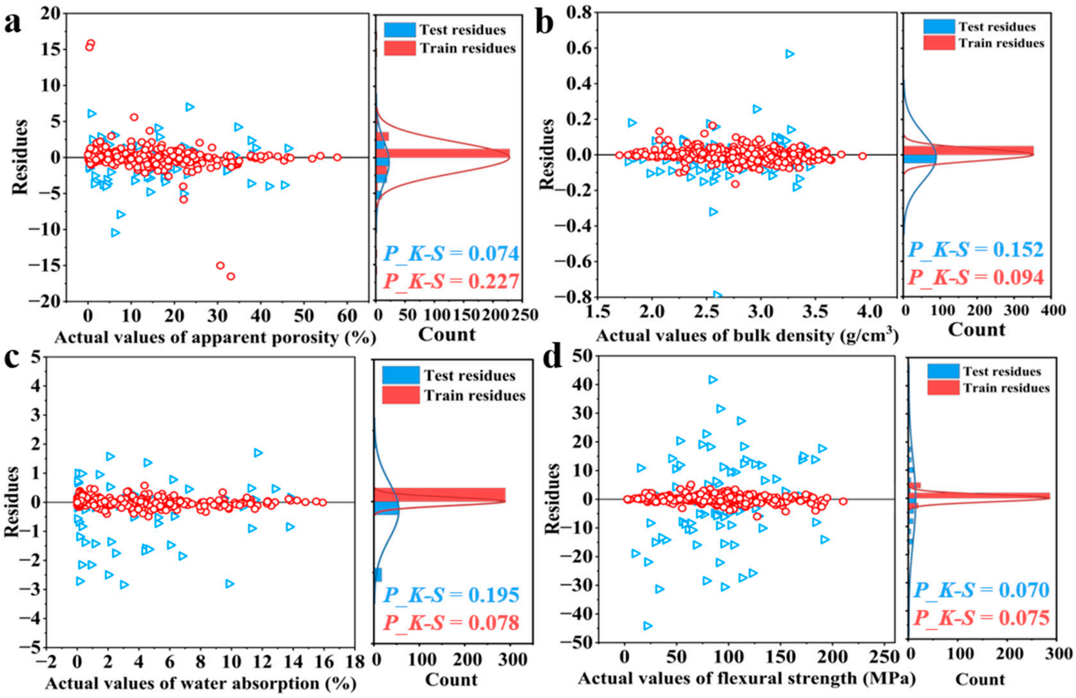

Figure S5. Residual distributions between predicted and actual values for (a) apparent porosity, (b) bulk density, (c) water absorption, and (d) flexural strength based on GBR models.

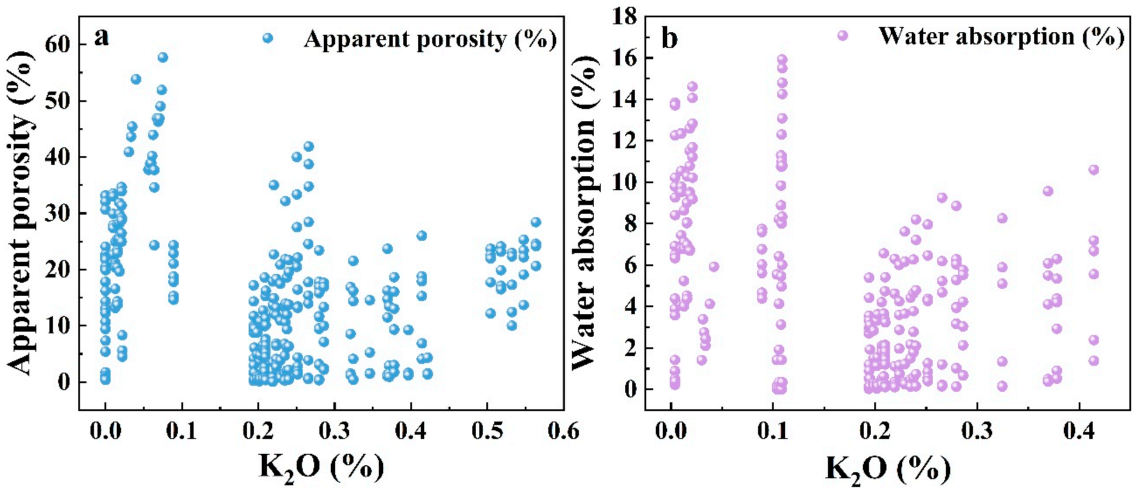

Figure S6. Effect of minor K_2O content on apparent porosity (a) and water absorption (b) based on inputs.

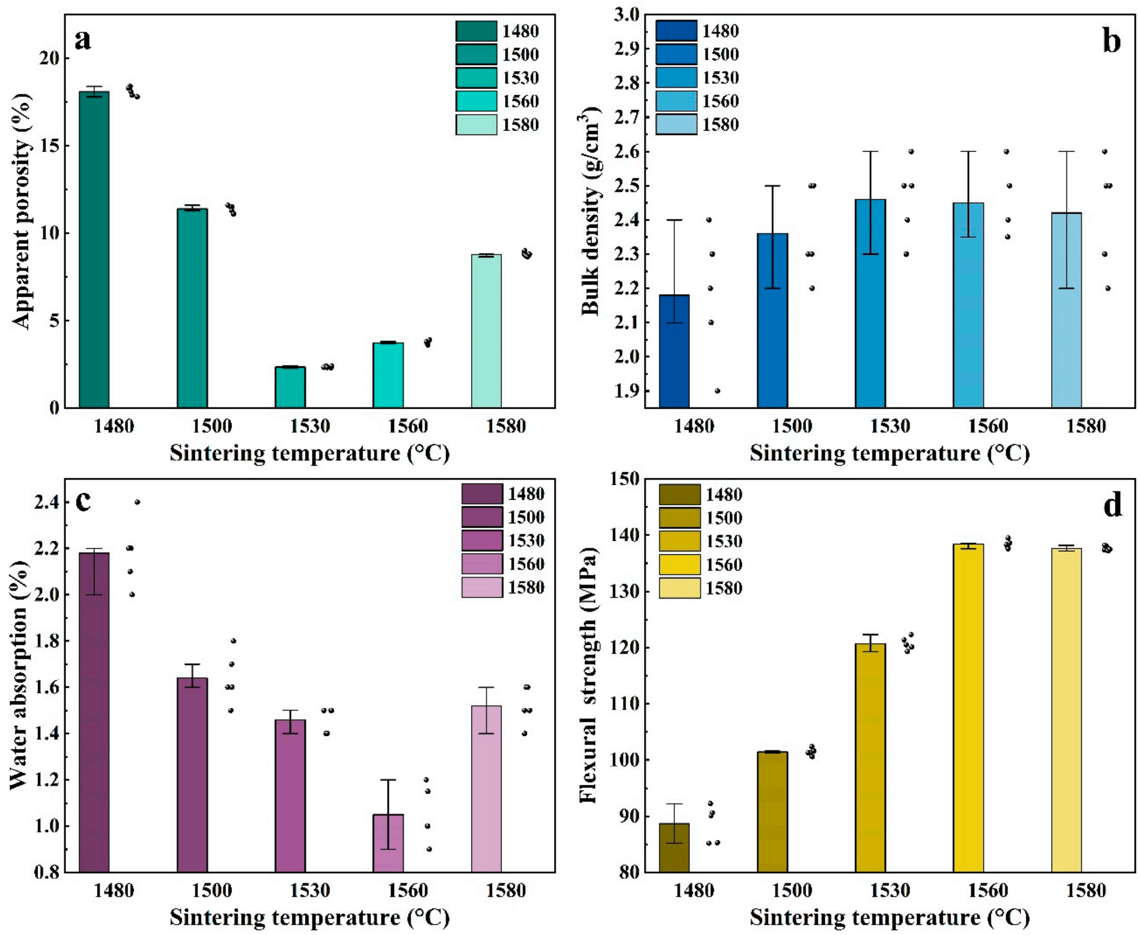

Figure S7. Experimental data of samples properties at different temperatures: apparent porosity (a), bulk density (b), water absorption (c), and flexural strength (d).

Table S1

Optimal hyper-parameter of ANN model for single-target ML prediction based on dataset #1~#4.

Dataset	Target (output)	Activation	Learning algorithm	Learning rate init	Hidden layer sizes
#1	apparent porosity (%)	Relu	Adam	0.001	(60, 50)
#2	water absorption (%)				(10, 60)
#3	bulk density (g/cm^3)				(64, 64)
#4	flexural strength (MPa)				(88, 64)

Table S2
Experimental validation results.

Items	Sintering temperature (°C)	Apparent porosity (%)			Bulk density (g/cm³)			Water absorption (%)			Flexural Strength (MPa)			Ref.
		Pv [a]	Ev [b]	Re [*] (%)	Pv [a]	Ev [b]	Re [*] (%)	Pv [a]	Ev [b]	Re [*] (%)	Pv [a]	Ev [b]	Re [*] (%)	
B0	1480	32.70	35.35	8.11	2.01	2.12	5.47	2.48	2.20	-11.28	80.50	61.46	-23.66	[1]
	1500	31.07	33.34	7.31	2.15	2.24	4.19	2.05	1.98	-3.26	75.56	65.70	-13.06	
	1530	20.07	24.72	23.17	2.05	2.14	4.39	2.01	2.04	1.24	97.73	73.12	-25.18	
	1560	15.70	18.20	15.94	2.13	2.16	1.37	2.19	2.16	-1.53	98.73	83.74	-15.19	
	1580	16.90	19.85	17.43	2.02	2.16	6.93	1.14	1.17	2.04	90.24	79.13	-12.32	
B1	1480	28.21	30.47	8.02	2.01	2.18	8.46	2.52	2.78	10.15	87.46	67.65	-22.65	
	1500	32.58	32.03	-1.66	2.15	2.19	1.86	2.04	2.09	2.25	105.52	75.42	-28.53	
	1530	20.03	20.54	2.55	2.06	2.11	2.79	1.79	1.76	-1.73	97.69	80.90	-17.19	
	1560	16.41	17.49	6.58	2.29	2.24	-2.42	0.98	1.07	9.65	98.86	95.93	-2.96	
	1580	17.62	18.37	4.23	2.18	2.22	1.79	1.09	1.05	-3.56	90.38	99.12	9.68	
B2	1480	28.17	31.08	10.33	2.05	2.11	2.88	2.52	2.11	-16.40	101.75	74.02	-27.26	
	1500	22.32	24.46	9.59	2.11	2.15	1.92	2.04	2.15	5.26	105.82	85.32	-19.37	
	1530	17.78	14.27	-19.70	2.15	2.28	6.08	1.50	1.68	12.14	97.99	99.49	1.53	
	1560	12.11	10.01	-17.30	2.33	2.36	1.41	1.08	1.16	7.62	98.99	107.44	8.54	
	1580	9.31	11.58	24.34	2.30	2.38	3.42	1.07	1.18	10.58	90.50	103.20	14.04	
B3	1480	18.68	21.68	16.09	2.12	2.17	2.36	2.84	2.66	-6.37	101.97	86.21	-15.45	
	1500	23.12	19.24	-16.79	2.30	2.32	0.78	2.31	2.32	0.32	106.03	99.65	-6.02	
	1530	3.58	4.09	14.25	2.29	2.40	4.56	1.71	1.50	-12.19	98.20	127.25	29.58	
	1560	1.49	1.39	-6.47	2.37	2.48	4.55	1.28	1.48	15.38	125.99	140.71	11.68	
	1580	5.12	4.78	-6.51	2.35	2.47	5.01	1.05	1.17	11.56	116.50	131.67	13.02	
B4	1480	18.37	18.10	-1.46	2.22	2.18	-1.85	2.27	2.18	-3.93	99.74	88.69	-11.08	This study
	1500	12.06	11.40	-5.48	2.29	2.36	3.03	1.79	1.64	-8.41	103.10	101.44	-1.61	
	1530	2.11	2.35	11.59	2.40	2.46	2.45	1.48	1.46	-1.26	111.49	120.72	8.27	
	1560	3.54	3.74	5.74	2.44	2.45	0.45	0.91	1.05	15.78	129.35	138.39	6.99	
	1580	7.57	8.79	16.16	2.42	2.42	0.17	1.60	1.52	-5.22	121.02	137.68	13.77	

[a] Predicted value

[b] Experimental value

[*] Calculation for relative error: $error_{Relative} = \frac{Pv-Ev}{Ev} \times 100\%$.

References

[1] Z. Liu, C. Wu, N. Xie, J. Zhu, Y. Liu, S. Huang, X. Shen, Z. Yang, X. Lin, L. Kong, Mechanical properties of in situ synthesized mullite-based composite ceramics with three-dimensional network structure, Int. J. Appl. Ceram. Technol. 19 (2022) 1659–1668. https://doi.org/10.1111/ijac.13982.
